# Case Report: Challenges in the Diagnosis of a Case of Mal de Meleda and a Therapeutic Attempt of Ixekizumab and Adalimumab

**DOI:** 10.3389/fmed.2022.821301

**Published:** 2022-03-10

**Authors:** Yuwei Dai, Xiaodong Zheng, Qi Zhang, Xia Hu, Peiguang Wang, Sen Yang

**Affiliations:** ^1^Department of Dermatology, The First Affiliated Hospital, Anhui Medical University, Hefei, China; ^2^Institute of Dermatology, Anhui Medical University, Hefei, China; ^3^Key Laboratory of Dermatology, Anhui Medical University, Ministry of Education, Hefei, China; ^4^Provincial Laboratory of Inflammatory and Immune Mediated Diseases, Hefei, China; ^5^Ferry Outpatient Department, The Ferry Skin Research Institute, Hefei, China

**Keywords:** Mal de Meleda, MDM, Exomiser, HPO terms, Ixekizumab, Adalimumab

## Abstract

**Background:**

Mal de Meleda (MDM, OMIM 248300) is an autosomal recessive disease characterized by symmetrical and progressive palmoplantar hyperkeratosis soon after birth. Mutations in *SLURP1* gene could lead to MDM. Clinically, MDM is easily misdiagnosed as other types of keratoderma due to phenotypic variation and overlap.

**Objective and Methods:**

A patient with suspected MDM was confirmed by the combination of next-generation sequencing and Exomiser, and the patient was attempted with the treatment of Ixekizumab and Adalimumab.

**Results:**

A homozygous mutation c.256G>A (p.Gly86Arg) in the *SLURP1* gene was identified in the patient. The inflammatory erythemas on his hands, feet and buttocks were mildly relieved after the treatment of high dose of Ixekizumab.

**Conclusions:**

Our findings helps to enhance the understanding of MDM. Ixekizumab may be a potential strategy to treat MDM.

## Introduction

Mal de Meleda (MDM, OMIM 248300) is a rare autosomal recessive inflammatory palmoplantar keratoderma with a prevalence of ~1 in 100,000. It appears in early infancy and features transgredient and progredient diffuse hyperkeratosis of the palms and soles, accompanied by hyperhidrosis. Perioral erythema, lichenoid plaques over the joints, and nail abnormalities are also usually documented (1, 2). The thick hyperkeratosis may cause scleroatrophic changes and even auto-amputation, which can severely impact function (3). Its typical histopathological changes were hyperkeratosis and acanthosis of the epidermis without epidermolysis, accompanied by infiltration of lymphocytes around the dermal blood vessels. Mutations in the SLURP1 gene, which encodes the secreted mammalian Ly-6/ urokinase-type plasminogen activator receptor (uPAR)-related protein-1(SLURP1), are implicated in the pathogenesis of MDM. SLURP1 is a participant of epidermal homeostasis, including growth, terminal differentiation, apoptosis, and cornification of keratinocytes (KCs) (4). Decreased expression of SLURP1 is also related to the occurrence of various epithelial malignancies (5).

Clinically, many dermatologists are not familiar with this rare disease. In addition, there is a phenotypic overlap between MDM and erythrokeratodermas and some types of palmoplantar keratoderma. So, it is very difficult to make the correct diagnosis according to the clinical manifestations of the MDM patients. The gene mutation analysis is helpful to confirm this disease.

Oral acitretin could be effective for MDM, but long-term use is not recommended because of side effects. Surgical treatment has also been reported (6). Here, we report a case of MDM diagnosed by the combination of next-generation sequencing and Exomiser and the efficacy of Ixekizumab and Adalimumab in the treatment of the patient.

## Report

**Figure 1:**
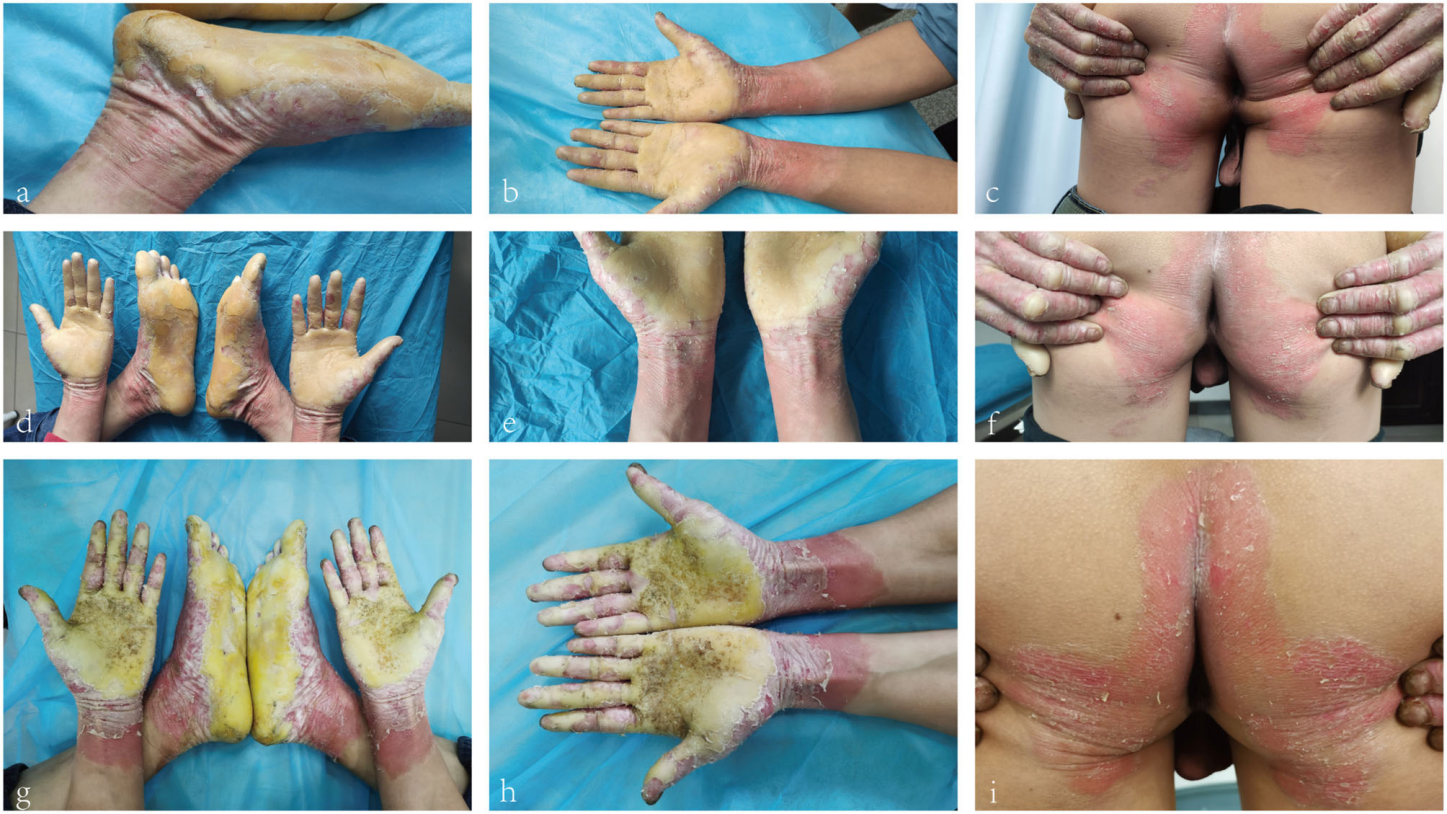
**(a–c)** Waxy, thick, pale-yellow scales on the palms and soles. Crimson erythema with a little scale on buttocks. **(d–f)** Retraction of erythema edge on the forearm and pale erythema on buttocks. **(g–i)** Crimson erythema on the forearm and buttocks. Increased area of forearm erythema.

**Figure 2:**
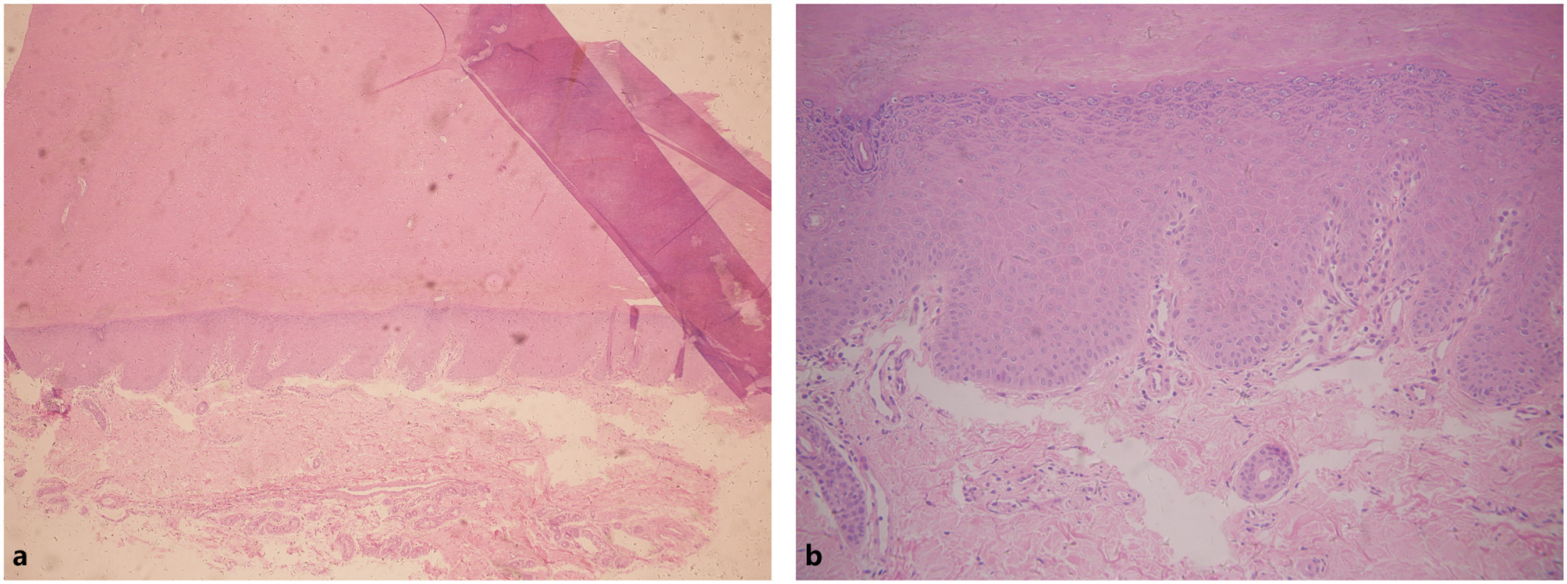
Hyperkeratosis in epithelial cells. Infiltration of a few inflammatory cells in superficial dermal. **(a)** Hyperkeratosis in epithelial cells. **(b)** Infiltration of a small number of inflammatory cells in superficial dermal.

A 26-year-old Chinese male presented with erythema and thick scales on his palms and soles. The skin lesions developed at about his 1 year old and then gradually deteriorated except a short spontaneous remission during his puberty. The symptoms were more severe in winter than in summer. The patient intermittently took oral retinoids and applied topical keratolytics. The long-term efficacy was not obtained. Other members did not have similar symptoms in his family. Upon cutaneous examination, waxy, thick, pale yellow scales was covered on the base of erythema in his palms and soles in a 'glove and socks' pattern. A large erythema with a little scale was present on the central area of his buttocks. His nails obviously became thick (Figures 1a–c). His general health was overall normal. Histology of the palmar lesion showed hyperkeratosis, proliferation of the overlying squamous epithelial cells and superficial dermal small blood vessels accompanied by a few inflammatory cells (Figure 2). There was no abnormal increase in the levels of cytokines IL-1B, IL-6, IL-8, IL-10, and IL-2R in his serum. Initially, we considered the diagnosis as progressive symmetric erythrokeratoderma (PSEK, MIM 602036) based on the patient's history, clinical and pathological manifestations. None of mutations in the related seven genes (GJB3, GJB4, GJA1, PERP, KRT83, KDSR, TRPM4) was found by DNA sequencing. Subsequently, we performed second-generation sequencing combined with the clinical phenotype database and analysis software, and eventually obtained the diagnosis of MDM. After the patient's consent, he was given 160 mg Ixekizumab for the first time. Two weeks later, the erythema and scales of his hands and feet were mildly reduced (Figures 1d–f). The second and third dose of injection (both 80 mg Ixekizumab) were administered at 2 weeks of interval. The patient's symptoms became worsened slightly after the third treatment. One month later, Adalimumab with dose of 40 mg was given subcutaneously. The patient showed more aggravated cutaneous lesions after 1 month (Figures 1g–i).

## Method and Materials

The peripheral blood of the patient and his parents were collected after obtaining their informed consent and the approval of the Ethics Committee of Anhui Medical University. Genomic DNA was extracted by DNA extraction kit (Promega, Madison, WI, United States).

### Whole Genome Sequencing

The qualified genomic DNA samples from three individuals were fragmented using Covaris technology, and 350 bp size fragments were selected. We gained the raw data after amplifying and sequencing (BGISEQ-500 platform) these fragments. Then we cleaned the raw data for mapping (Burrows-Wheeler Aligner V0.7.15) and realignment. Subsequently, we used Genome Analysis Toolkit (GATK, v3.3.0) to detect SNPs and InDels. Finally, we annotated the SNPs and InDels by the SnpEff tool (http://snpeff.sourceforge.net/SnpEff_manual.html).

HPO terms were obtained by entering the features of the patient's phenotype to Phenomizer (http://compbio.charite.de/phenomizer/) (Table 1).

**Table 1:** Detailed descriptions of clinical abnormalities.

HP:0010783	Erythema
HP:0001019	Erythroderma
HP:0000988	Skin rash
HP:0001231	Abnormality of the fingernails
HP:0000958	Dry skin
HP:0005595	Generalized hyperkeratosis
HP:0007390	Hyperkeratosis with erythema
HP:0000982	Palmoplantar keratoderma
HP:0007548	Palmoplantar keratosis with erythema and scale
HP:0001036	Parakeratosis
HP:0005588	Patchy palmoplantar keratoderma
HP:0007501	Streaks of hyperkeratosis along each finger onto the palm
HP:0004322	Short stature

We put the three VCF files (WGS) and HPO Terms into Exomiser (http://www.sanger.ac.uk/science/tools/exomiser) to discover the disease-causing mutation.

### Whole-Exome Sequencing

To replicate our WGS findings, we subsequently performed whole exome sequencing for the proband and 10 healthy controls. We chose 150–250 bp size fragments of the qualified genomic DNA sample of the patient randomly. The subsequent analysis flow was the same as WGS except that we used GATK (v.3.7) to call out variants.

## Results

**Figure 3:**
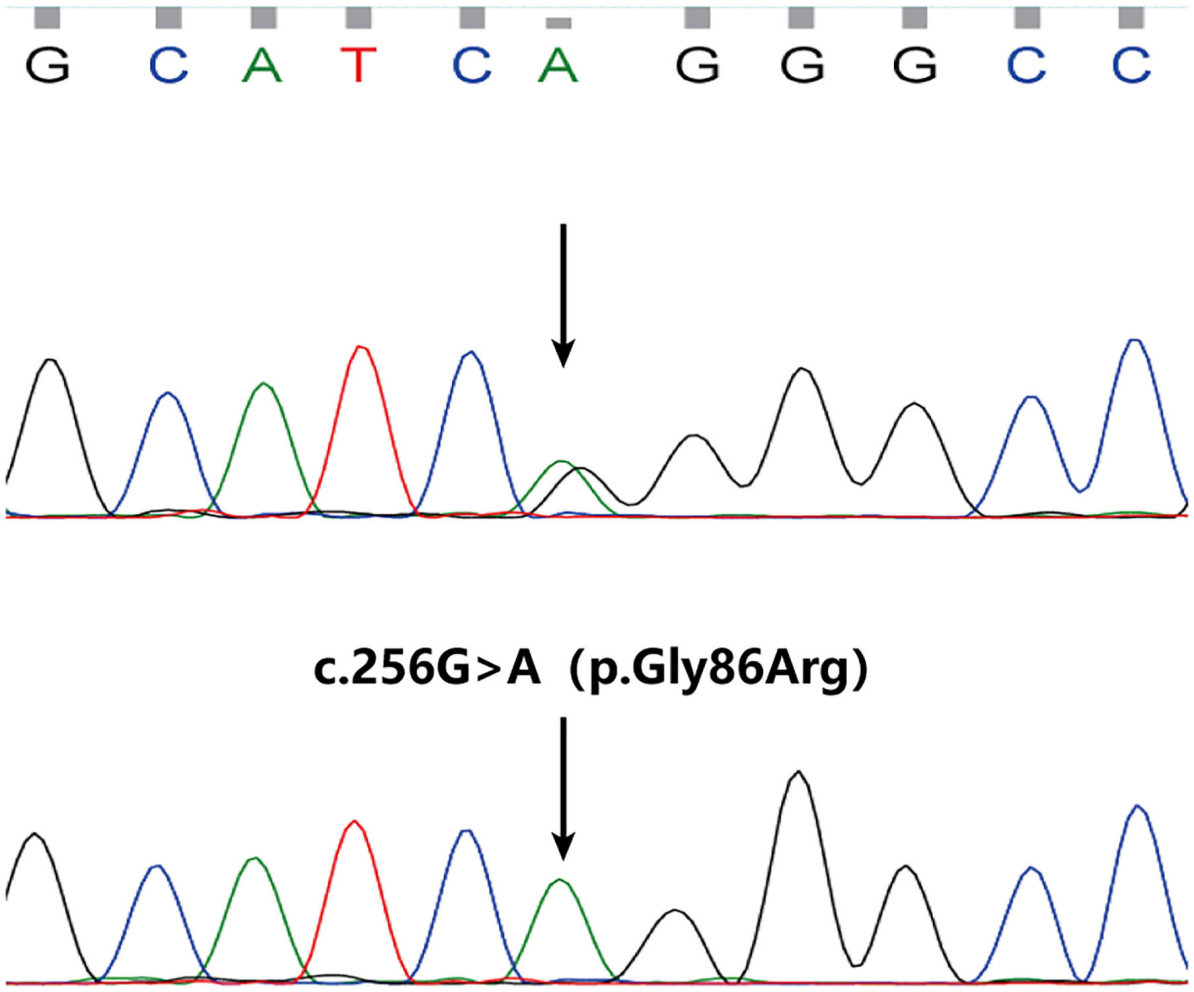
A homozygous mutation in SLURP1 identified in the proband (the lower) and sequencing result of his parents (normal, the upper).

Among WGS data of 3 persons, we called out 6,267,332 sites, removing Polymorphism loci and remaining 5,858,568 dimorphism loci. A total of 203,673 sites were called out from 11 persons' WES data, and there were 186,634 dimorphism loci. From Exomiser results, a homozygous mutation c.256G>A(NM_020427.3) in exon 3 of *SLURP1* gene got the highest score of 0.973 and was predicted to result in an amino acid substitution of p.Gly86Arg. The mutation has been previously reported in Asian patients (7). Sanger sequencing revealed the mutation perfectly co-segregated with the phenotype (Figure 3). Therefore, the patient was diagnosed as MDM according to his clinical features and the finding of *SLURP1* gene mutation analysis.

## Discussion

PSEK (Progressive Symmetrical Erythrokeratoderma, OMIM 133200) is inherited in the recessive or dominant mode, featuring symmetric and progressive erythematous hyperkeratotic patches over the body, particularly on the trunk and limbs, buttocks and face. Approximately 50% of affected individuals develop palmoplantar keratoderma (8, 9). Although PSEK, like MDM, often develops shortly after birth, the former relieves after a brief exacerbation in adolescence, while the latter has lifelong symptoms that tend to worsen with age. In addition, the perioral erythema was not obvious in the patient, which made us ignore this symptom during physical examination.

Wild-type SLURP1 is significant for normal T-cell activation (10). The mutation we observed is in a highly conserved region, resulting in partial SLURP1 inactivation. Diminished SLURP1 activity affects the regulation of epidermal homeostasis and immune function, leading to inflammatory and proliferative skin lesions (4, 11). These MDM mechanisms suggest that pharmacological regulation of epidermal immunity and inflammatory cytokines may be feasible. In addition, Moriwaki et al. found that SLURP1 is involved in the pathophysiology of psoriasis by regulating proliferation and differentiation of keratinocytes (12). This led us to wonder whether the inflammatory pathways in the skin of MDM patients overlap with those of psoriasis patients. The patient was treated by Ixekizumab on the basis of the conjecture. Currently, Ixekizumab is one of the mainstream drugs for the treatment of moderate to severe psoriasis, which selectively binds to IL-17A and inhibits binding with IL-17R, and then inhibits the secretion of pro-inflammatory cytokines and chemokines by targeting cells with downstream effects on cellular elements. The patient showed slight improvement in erythema after the use of high dose of Ixekizumab, suggesting that IL-17A is possibly involved in the development of MDM. However, there was no obvious improvement and even some exacerbation after the patient was treated with the recommended dose according to the standard protocol of Ixekizumab.

The patient required to change the treatment considering poor efficacy and costly expense of Ixekizumab. So, we selected another type of biological agents commonly used in psoriasis, Adalimumab. It is a TNF-α antagonist approved for the treatment of rheumatoid arthritis, ankylosing spondylitis, moderate to severe psoriasis vulgaris and psoriasis arthritis (13). A previous study has demonstrated SLURP1 is capable of inhibiting TNF-α from release by macrophages (14). Besides, excessive expression of TNF-α was observed within the whole layers of epidermis in an MDM patient with mutation c.256G>A (15). Ertle et al. (16) found the beneficial effects of SLURP1 in suppressing the TNF-a-induced upregulation of inflammatory cytokines. These findings indicate that the over expression of TNF-α in skin may be related to the pathogenesis of MDM. However, the patient in our study showed poor response to the treatment of Adalimumab.

In summary, the diagnosis of MDM is challenging. IL-17A is likely to participate in the inflammatory mechanism of MDM. Possibly, it is beneficial to control symptoms of MDM for high dose of Ixekizumab. Unfortunately, we did not carry out some investigation with regard to the expression of IL-17A and TNF-α in the lesional skin before treatment. Anyhow, the use of biological agents is a meaningful attempt in the treatment of MDM despite poor efficacy for the patient in our study.

## Data Availability Statement

The datasets presented in this study can be found in online repositories. The names of the repository/repositories and accession number(s) can be found below: https://www.ncbi.nlm.nih.gov/sra/PRJNA788551.

## Ethics Statement

The studies involving human participants were reviewed and approved by the Ethics Committee of Anhui Medical University. The patients/participants provided their written informed consent to participate in this study. Written informed consent was obtained from the individual(s) for the publication of any potentially identifiable images included in this article.

## Author Contributions

YD and XZ conducted WGS, WES, and Sanger sequencing. YD wrote the manuscript. QZ and XH collected clinical data and blood samples and performed DNA extraction. PW and SY were responsible for the study design and guiding of the study implementation and revised the manuscript. All authors contributed to the article and approved the submitted version.

## Funding

The work was supported by the Science and Technology Action Plans for the Prevention and Treatment of Major Diseases sponsored by National Health and Family Planning Commission of the People's Republic of China (Grant No. 2017ZX-01E-002).

## Conflict of Interest

The authors declare that the research was conducted in the absence of any commercial or financial relationships that could be construed as a potential conflict of interest.

## Publisher's Note

All claims expressed in this article are solely those of the authors and do not necessarily represent those of their affiliated organizations, or those of the publisher, the editors and the reviewers. Any product that may be evaluated in this article, or claim that may be made by its manufacturer, is not guaranteed or endorsed by the publisher.
