# Corrigendum: Case report: Challenges in the diagnosis of a case of Mal de Meleda and a therapeutic attempt of ixekizumab and Adalimumab

**DOI:** 10.3389/fmed.2022.1106530

**Published:** 2022-12-12

**Authors:** Yuwei Dai, Xiaodong Zheng, Qi Zhang, Xia Hu, Peiguang Wang, Sen Yang

**Affiliations:** ^1^Department of Dermatology, The First Affiliated Hospital, Anhui Medical University, Hefei, China; ^2^Institute of Dermatology, Anhui Medical University, Hefei, China; ^3^Key Laboratory of Dermatology, Anhui Medical University, Ministry of Education, Hefei, China; ^4^Provincial Laboratory of Inflammatory and Immune Mediated Diseases, Hefei, China; ^5^Ferry Outpatient Department, The Ferry Skin Research Institute, Hefei, China

**Keywords:** Mal de Meleda, MDM, Exomiser, HPO terms, Ixekizumab, Adalimumab

In the published article, there was an error in the dosage of adalimumab. A correction has been made to **Report**, 1. This sentence previously stated:

“One month later, Adalimumab with dose of 300 mg was given subcutaneously.”

The corrected sentence appears below:

“One month later, Adalimumab with dose of 40 mg was given subcutaneously.”

The authors apologize for this error and state that this does not change the scientific conclusions of the article in any way. The original article has been updated.

