# Supplementary figures and images for: Case Report: Challenges in the Diagnosis of a Case of Mal de Meleda and a Therapeutic Attempt of Ixekizumab and Adalimumab
Source: Front Med (Lausanne). 2022 Mar 10;9:821301. doi: 10.3389/fmed.2022.821301 (PMC8961326; doi:10.3389/fmed.2022.821301)

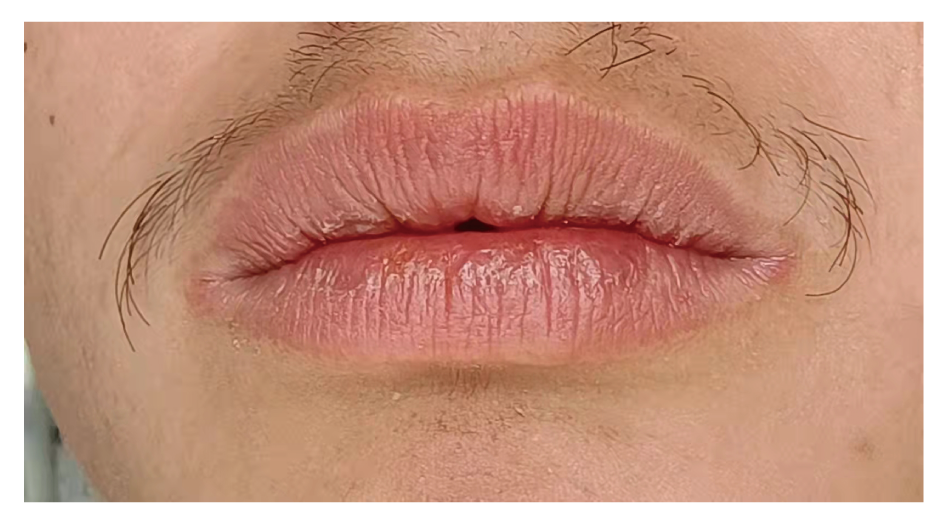

Supplement: Supplementary file 1 [file Image_1.TIF]
